# Generalizability of machine learning for classification of schizophrenia based on resting‐state functional MRI data

**DOI:** 10.1002/hbm.24797

**Published:** 2019-10-01

**Authors:** Xin‐Lu Cai, Dong‐Jie Xie, Kristoffer H. Madsen, Yong‐Ming Wang, Sophie Alida Bögemann, Eric F. C. Cheung, Arne Møller, Raymond C. K. Chan

**Affiliations:** ^1^ Neuropsychology and Applied Cognitive Neuroscience Laboratory, CAS Key Laboratory of Mental Health Institute of Psychology Beijing China; ^2^ Sino‐Danish College, University of Chinese Academy of Sciences Beijing China; ^3^ Sino‐Danish Center for Education and Research Beijing China; ^4^ Hangzhou College of Preschool Teacher Education Zhejiang Normal University Hangzhou China; ^5^ Danish Research Centre for Magnetic Resonance, Centre for Functional and Diagnostic Imaging and Research Copenhagen University Hospital Hvidovre Copenhagen Denmark; ^6^ Department of Applied Mathematics and Computer Science Technical University of Denmark Kongens Lyngby Denmark; ^7^ Castle Peak Hospital, Hong Kong Special Administrative Region China; ^8^ Department of Nuclear Medicine and PET Centre Aarhus University Hospital Aarhus Denmark; ^9^ Department of Psychology University of Chinese Academy of Sciences Beijing China

**Keywords:** generalizability, machine learning, reproducibility, schizophrenia spectrum disorders

## Abstract

Machine learning has increasingly been applied to classification of schizophrenia in neuroimaging research. However, direct replication studies and studies seeking to investigate generalizability are scarce. To address these issues, we assessed within‐site and between‐site generalizability of a machine learning classification framework which achieved excellent performance in a previous study using two independent resting‐state functional magnetic resonance imaging data sets collected from different sites and scanners. We established within‐site generalizability of the classification framework in the main data set using cross‐validation. Then, we trained a model in the main data set and investigated between‐site generalization in the validated data set using external validation. Finally, recognizing the poor between‐site generalization performance, we updated the unsupervised algorithm to investigate if transfer learning using additional unlabeled data were able to improve between‐site classification performance. Cross‐validation showed that the published classification procedure achieved an accuracy of 0.73 using majority voting across all selected components. External validation found a classification accuracy of 0.55 (not significant) and 0.70 (significant) using the direct and transfer learning procedures, respectively. The failure of direct generalization from one site to another demonstrates the limitation of within‐site cross‐validation and points toward the need to incorporate efforts to facilitate application of machine learning across multiple data sets. The improvement in performance with transfer learning highlights the importance of taking into account the properties of data when constructing predictive models across samples and sites. Our findings suggest that machine learning classification result based on a single study should be interpreted cautiously.

## 1 INTRODUCTION

Schizophrenia is a serious mental disorder that imposes a significant burden on society around the world (Charlson, Baxter, Cheng, Shidhaye, & Whiteford, 2016). The clinical symptoms of schizophrenia are heterogeneous (Arango & Carpenter, 2011; Owen, 2014), and its diagnosis is still dependent on the subjective report from patients and assessment by clinicians (Frances, 1994).

With the aim to provide objective assessment to guide clinical practice, integrative psychobiological approaches including the Research Domain Criteria initiative (Kozak & Cuthbert, 2016) and the Hierarchical Taxonomy of Psychopathology (Kotov et al., 2017) suggest breaking the symptom‐oriented approach down to different levels of neuroscientific analysis for mental disorders. In line with these new approaches, patients with schizophrenia have been demonstrated to have functional and structural brain alterations from magnetic resonance imaging (MRI) research (Cheng, Newman, et al., 2015; Cheng, Palaniyappan, et al., 2015; Ellison‐Wright & Bullmore, 2009; Haijma et al., 2013). Among the various MRI techniques, resting‐state functional MRI (rsfMRI) has been widely applied and aberrant brain activity has been reported in schizophrenia patients (Cheng, Newman, et al., 2015; Cheng, Palaniyappan, et al., 2015; Tang et al., 2013; Xu & Lipsky, 2014).

However, results from traditional MRI studies are group‐level data and have limited clinical applications. More recently, machine learning has been increasingly utilized to optimize the use of brain imaging data in clinical classification and build predictive models for individualized diagnosis for different psychiatric disorders (Arbabshirani, Plis, Sui, & Calhoun, 2017). Machine learning is defined as the process of enabling computers to acquire the ability to learn patterns of data without being explicitly programmed to do so (Samuel, 1959). Multivariate machine learning approaches are algorithms specialized in recognizing patterns from high‐dimensional data like brain imaging data, which can capture the complex relationships between brain regions compared with univariate methods (Davatzikos, 2004). The general machine learning framework with rsfMRI data for computational psychiatry includes: (a) preprocessing the imaging data; (b) separating the training data and the testing data completely; (c) extracting and selecting features from the training data; (d) training the predictive model; and (e) generalizing the predictive model to the testing data. In this framework, assessing generalizability is one of the most important steps in evaluating predictive models, which simulates the real‐world context. Generalizability is assessed in two distinct settings: within‐site and between‐site. Two main strategies, internal validation (or cross‐validation) and external validation, have been used to assess generalizability. Within‐site generalizability is typically established using cross‐validation. In this setting, a data set collected from a single site is repeatedly split into independent training and test data sets, and the performance of the model is assessed in the training set for each split. However, to assess between‐site generalizability, the predictive model is trained on a data set from one site and then applied to an independent data set collected in a separate site using external validation. Using internal validation is practical considering the difficulty in collecting data from different sites, but it could lead to an overestimation of performance due to overfitting of the predictive model to one specific data set, compared with external validation which considers generalizability across different data sets (Woo, Chang, Lindquist, & Wager, 2017). It is therefore important to assess generalizability using both internal and external validation methods.

A number of promising studies have successfully classified patients with schizophrenia from healthy controls based on rsfMRI data using machine learning approaches. As shown in Table 1, accumulated studies have utilized machine learning as a tool to analyze rsfMRI data, investigate the underlying neural mechanisms, recognize specific brain patterns, and classify patients with schizophrenia from healthy controls at the individual level with accuracies ranging from 65 to 95% (Arbabshirani, Kiehl, Pearlson, & Calhoun, 2013; Cao et al., 2018; Cheng, Newman, et al., 2015; Cheng, Palaniyappan, et al., 2015; Du et al., 2012; Venkataraman et al., 2012). However, most of these studies have only assessed generalizability using internal validation methods. While two studies have used external validation to assess the generalizability of their classification methods, they did so in independent rsfMRI data sets (Cui, Liu, Song, et al., 2018; Cui, Liu, Wang, et al., 2018; Skatun et al., 2017) and no study has independently replicated the machine learning procedure from a previous study.

**Table 1:** Summary of machine learning studies in schizophrenia based on rsfMRI

Reference	Participants	Feature	Feature extraction	Classifier	Accuracy (%)
Shen, Wang, Liu, and Hu (2010)	SZ = 32, HC = 20	FC among 116 regions	Correlation coefficient rank; LLE	C‐means clustering	86.5
Fan et al. (2011)	SZ = 31, HC = 31	Functional brain networks from ICA	ICA, Grassmann manifold analysis	SVM	85.5
Du et al. (2012)	SZ = 28, HC = 28	Spatial components from ICA	*t* test; PCA; FLD	Majority voting	93
Tang, Wang, Cao, and Tan (2012)	SZ = 22, HC = 22	FC by 90 regions	Correlation coefficient rank; PCA	Linear SVM	93.2
Bassett, Nelson, Mueller, Camchong, and Lim (2012)	SZ = 15, HC = 14	FC by the graph	No	Linear kernel SVM	75
Venkataraman, Whitford, Westin, Golland, and Kubicki (2012)	SZ = 18, HC = 18	FC by 77 ROIs	Select by prior knowledge and random forest	Majority voting	75
Anderson and Cohen (2013)	SZ = 72, HC = 74	FNC by the graph	ICA	SVM	65
Arbabshirani et al. (2013)	SZ = 28, HC = 28	FNC	ICA; visually inspect	K‐nearest neighbors	96
Fekete et al. (2013)	SZ = 8, HC = 10	FNC by the graph	No	Multi kernel block diagonal optimization	100
Su, Wang, Shen, Feng, and Hu (2013)	SZ = 32, HC = 32	FC	Correlation coefficient rank	Linear kernel SVM	81.2
Watanabe, Kessler, Scott, Angstadt, and Sripada (2014)	SZ = 54, HC = 67	FC by 347 ROIs	Elastic‐net	SVM	73.5
Cheng, Newman, et al. (2015) and Cheng, Palaniyappan, et al. (2015)	SZ = 415, HC = 405	FC by BWAS	No	SVM	75.8
Chyzhyk, Savio, and Grana (2015)	SZ = 74, HC = 72	FC/ local activity	Pearson's correlation coefficient	SVM	91.2/100
Savio and Grana (2015)	SZ = 72, HC = 74	ALFF, fALFF, VMHC, ReHo	Voxel site saliency measures	SVM; RF	80
Cheng, Newman, et al. (2015) and Cheng, Palaniyappan, et al. (2015)	SZ = 27, HC = 36	BC of FC	Rank BC	SVM	79
Kaufmann et al. (2015)	SZ = 71, HC = 196	FC from ICA	No	Regularized LDA	84.4
Kim et al. (2016)	SZ = 50, HC = 50	FC among 116 regions	No	Deep neural network	86
Skatun et al. (2017)	SZ = 182, HC = 348	FC from ICA	No	Regularized LDA	78.3
Cui, Liu, Song, et al. (2018) and Cui, Liu, Wang, et al. (2018)	SZ = 108, HC = 121	FC by 90 ROIs	Two sample *t* tests, LASSO	SVM	82.6
Cao et al. (2018)	SZ = 43, HC = 29	MI and FC between STC and other cortical regions	Top 10 features	SVM	78.6

Abbreviations: BC, betweenness centrality; BWAS, brain‐wide association study; FLD, Fisher linear discriminant; FNC, functional network connectivity; HC, healthy controls; LASSO, least absolute shrinkage and selection operator; LDA, linear discriminative analysis; LLE, locally linear embedding; MI, mutual information; PCA, principle component analysis; RF, random forest; ROI, region of interest; STC, superior temporal cortex; SZ, patients with schizophrenia; VMHC, voxel‐mirrored homotopic connectivity.

In this study, the overall aim was to assess the within‐site and between‐site generalizability of a previous machine learning framework of rsfMRI data that have shown promising performance using both internal and external validation methods. Among the various previous studies, Du et al. (2012) have reported an excellent classification accuracy of 0.93 using rsfMRI data and 0.98 using fMRI data in identifying schizophrenia patients from healthy controls based on their own machine learning procedure. Therefore, we examined the generalizability of this machine learning procedure in the present study. We first investigated the generalizability of the classification procedure in a main data set from a single site (internal validation) following the exact steps from Du et al.'s study. Then, we assessed the between‐site generalizability to a completely independent data set (validated data set) from a different site (external validation) to test whether factors from different sites, such as scanning setting and procedure, would influence generalizability. To further explore the generalizability of the procedure rather than the algorithm itself, we updated the unsupervised part of the algorithm to investigate the degree to which the performance of between‐site generalizability could be improved in the new setting. Importantly, the labels (schizophrenia or healthy control) of the testing data set were not used when training the predictive model in the last step, which served to determine the between‐site generalizability of the procedure from independent data sets. Given the excellent performance of the procedure reported by Du et al. (2012), we hypothesized that this machine learning procedure could discriminate patients with schizophrenia from healthy controls with promising classification performance with good within‐site generalizability. Due to the effect of site on rsfMRI data (Dansereau et al., 2017), we further hypothesized that the cross‐site generalizability of the procedure would be compromised. Moreover, because some site effects could be taken into account when updating the unsupervised last step of the procedure, we further hypothesized that the between‐site generalizability would improve in the new setting.

## 2 METHODS

### 2.1 Participants

Two data sets of patients with schizophrenia and healthy controls were included in this study. In the main data set, 51 patients with schizophrenia and 51 healthy controls were recruited (Table 2). Schizophrenia patients were recruited from the Community Health Service Centre of the Institute of Mental Health (the sixth Affiliated Hospital of Peking University) in the Haidian District of Beijing, China. In the validated data set, 34 patients with schizophrenia and 27 healthy controls were recruited (Table 2). The patients with schizophrenia were recruited from the Changsha Psychiatric Hospital, Changsha, Hunan province, China. The diagnosis of schizophrenia was ascertained with the Structural Clinical Interview for the Diagnostic and Statistical Manual of Mental Disorders (DSM‐IV), Fourth Edition (Frances, 1994). The Positive and Negative Syndrome Scale (PANSS) (Kay, Fiszbein, & Opler, 1987) was used to assess the severity of schizophrenia symptoms. Both the clinical assessment and the diagnostic interviews were conducted by experienced psychiatrists. All patients were taking antipsychotic medications. Healthy controls were recruited from the local community via advertisements. They had no personal and family history of mental disorders. Participants with neurological disorders, substance abuse, and/or dependence or head injuries were excluded. The short form of the Chinese version of the Wechsler Adult Intelligence Scale (Gong, 1992) was used to estimate the IQ of all participants.

**Table 2:** Demographic and clinical information of two data sets

	HC	SZ	*t*/χ ^2^	*p*
The main data set				
Demographics				
Age (years)	42.04 (12.165)	43.22 (10.885)	−.515	.608
Gender (male%)	35.29%	41.18%	.374	.684
Education (years)	12.80 (3.731)	12.07 (2.946)	1.105	.272
Estimated IQ*	119.76 (11.735)	107.68 (14.215)	4.663	.000
Clinical characteristics				
Onset age (years)		25.41 (9.185)		
Course (years)		16.66 (8.067)		
PANSS total		51.69 (14.771)		
PANSS positive		11.63 (4.858)		
PANSS negative		13.39 (5.783)		
PANSS general		26.67 (7.618)		
CPZ equivalent dose (mg)		236.56 (172.208)		
The validated data set				
Demographics				
Age* (years)	27.37 (7.344)	36.5 (7.140)	−4.898	.000
Gender* (male%)	44.44%	82.35%	9.580	.003
Education* (years)	13.93 (2.814)	12.18 (2.208)	2.722	.009
Estimated IQ	108.00 (20.196)	93.94 (24.799)	1.970	.055
Clinical characteristics				
Onset age (years)		24.17 (6.644)		
Course (years)		10.54 (7.868)		
PANSS total		67.72 (12.378)		
PANSS positive		10.00 (3.873)		
PANSS negative		23.16 (3.118)		
PANSS general		30.84 (7.238)		
CPZ equivalent dose (mg)		287.05 (193.830)		

*Note*. Table values: mean (*SD*).

Abbreviations: CPZ: chlorpromazine; HC, healthy controls; PANSS, the Positive and Negative Syndrome Scale; SZ, patients with schizophrenia.

*p < 0.05.

In addition, independent sample *t* tests were conducted to compare age, length of education and estimated IQ between the two healthy control groups (Table 3), and the same were conducted to compare onset age, duration of illness and severity of psychotic symptoms between the two patient groups. Chi‐square tests were also used to compare the gender proportion between the two healthy control groups and the two clinical groups. Finally, a five‐factor model of the PANSS (Anderson et al., 2017; Lindenmayer, Grochowski, & Hyman, 1995) comprising negative, positive, disorganized, excited, and anxiety symptom domains was computed and compared between the two clinical groups (Table 3).

**Table 3:** Comparison of demographic and clinical information between two sites

	SZ				HC			
	Main	Validated	*t*/χ^2^	*p*	Main	Validated	*t*/χ^2^	*p*
Demographics								
Age (years)	43.22 (10.885)	36.50 (7.14)	3.168	.002	42.04 (12.165)	43.22 (10.885)	−.515	.608
Gender (male%)	41.18%	82.35%	14.167	.000	35.29%	44.44%	.625	.470
Education (years)	12.07 (2.946)	12.18 (2.208)	−.182	.856	12.80 (3.731)	12.07 (2.946)	1.105	.272
Estimated IQ	107.68 (14.215)	93.94 (24.799)	3.206	.002	119.76 (11.735)	107.68 (14.215)	4.663	.000
Clinical characteristics								
Onset age (years)	25.41 (9.185)	24.17 (6.644)	.594	.554				
Course (years)	16.66 (8.067)	10.54 (7.868)	3.070	.003				
PANSS total	51.69 (14.771)	67.72 (12.378)	−4.678	.000				
PANSS positive	11.63 (4.858)	10.00 (3.873)	1.461	.148				
PANSS negative	13.39 (5.783)	23.16 (3.118)	−7.884	.000				
PANSS general	26.67 (7.618)	30.84 (7.238)	−2.28	.025				
CPZ equivalent dose (mg)	236.56 (172.208)	287.05 (193.830)	−1.024	.310				
PANSS_5_ negative	15.20 (6.636)	28.17 (4.428)	−8.544	.000				
PANSS_5_ positive	10.47 (4.888)	8.17 (2.973)	2.127	.037				
PANSS_5_ disorganized	15.22 (4.500)	17.09 (4.067)	−1.704	.093				
PANSS_5_ excited	5.61 (2.155)	5.92 (2.701)	−.533	.596				
PANSS_5_ anxiety	8.75 (3.918)	8.76 (3.358)	−.016	.987				

*Note*. Table values: mean (*SD*).

Abbreviations: CPZ, chlorpromazine; HC, healthy controls; main, main data set; PANSS, the Positive and Negative Syndrome Scale; PANSS_5_, the symptom domains calculated by the five‐factor model; SZ, patients with schizophrenia; validated: validated data set.

The study was approved by the Ethics Committee of the Institute of Psychology, the Chinese Academy of Sciences. All participants gave written informed consent.

### 2.2 Image acquisition

For the main data set, all participants were scanned in a 3‐T Siemens Tim Trio scanner at the Chaoyang Hospital, Beijing, China. The rsfMRI data were collected by an echo‐planar imaging (EPI) sequence utilizing gradient echo. Slices were acquired in interleaved order and the data consisted of 200 whole‐brain volumes (repetition time (TR) = 2,500 ms, echo time (TE) = 21 ms, flip angle = 90°, slice number = 42, slice thickness = 3.5 mm, matrix size = 64 × 64, field of view (FOV) = 200 mm, and voxel size = 3.1 × 3.1 × 3.5 mm^3^). T1‐weighted structural image data were collected for anatomical reference using a 3D magnetization‐prepared rapid gradient‐echo (MPRAGE) sequence (TR = 2,530 ms, TE = 2.34 ms, flip angle = 7°, FOV = 256 mm, slice thickness = 1 mm, slice number = 176, in‐plane matrix resolution = 256 × 256, and voxel size = 1 × 1 × 1 mm^3^).

For the validated data set, all participants were scanned in a 3‐T Siemens TIM Trio scanner in the Hunan Provincial People's Hospital, Changsha, Hunan province, China. The rsfMRI data were collected by an EPI sequence utilizing gradient echo. Slices were acquired in interleaved order and the data consisted of 180 whole‐brain volumes (TR = 2,000 ms, TE = 25 ms, flip angle = 80°, slice number = 32, slice thickness = 4.5 mm, matrix size = 64 × 64, FOV = 240 mm, and voxel size = 3.75 × 3.75 × 4.5 mm^3^). T1‐weighted structural image data were collected using a 3D MPRAGE sequence (TR = 2,000 ms, TE = 2.26 ms, flip angle = 8°, FOV = 256 mm, slice thickness = 1 mm, slice number = 176, in‐plane matrix resolution = 256 × 256, and voxel size = 1 × 1 × 1 mm^3^).

While participants were in the scanner, they were asked to remain as stationary as possible and their heads were stabilized with foam pads.

### 2.3 Data preprocessing

Image preprocessing was conducted with the Statistical Parameter Mapping (SPM) software (SPM12; http://www.fil.ion.ucl.ac.uk/spm/software/spm12), Data Processing Assistant for Resting‐State fMRI (DPARSF) Software (DPARSF4.3; http://rfmri.org/DPARSF), and a plugin‐in for SPM named Temporal Filter (http://www.brain-fmri.com/tempfilter) (Madsen, Krohne, Cai, Wang, & Chan, 2018). The two data sets were preprocessed separately with the same steps. To avoid T1 relaxation effects prior to equilibrium, the first five volumes of each resting state scan were excluded. Then, the remaining volumes for each of the functional imaging sessions were processed using DPARSF, including slice timing, realignment, and coregistration to the structural images. Mean frame‐wise displacement (FD) was calculated (Power, Barnes, Snyder, Schlaggar, & Petersen, 2012) for each participant. The percentage of time points with mean FD power >0.5 was lower than 10% for all participants. In order to remove nonstationary signals and other artifacts, the temporal filter toolbox (Madsen et al., 2018) was used. Processing steps included: despiking (Patel et al., 2014), high‐pass filtering and removal of low‐frequency drifts and motion by a 24‐parameter autoregressive model using realignment regressors and scrubbing (using a 1 mm relative movement threshold and a 1% DVARS threshold (Power et al., 2012)) (Friston, Williams, Howard, Frackowiak, & Turner, 1996). Subsequently, spatial normalization with DARTEL was adopted to normalize the functional images into Montreal Neurological Institute (MNI) space (Ashburner, 2009) in SPM12. Finally, an 8‐mm full‐width‐at‐half‐maximum isotropic Gaussian kernel was used to smooth the functional images (Table 5).

**Table 4:** Selected spatial components used for classification

Index	Component name	Spatial location	Peak MNI region	Peak MNI coordinates (mm)
(1)	Occipital lateral cortex	Left calcarine sulcus + left middle occipital gyrus + lingual gyrus	Left calcarine sulcus	−12, −93, −3
(2)	Occipital medial cortex	Calcarine	Left calcarine sulcus	0, −75, 9
(3)	Cerebellum	Left lobule VIII, left crus I, and lobule VI of cerebellar hemisphere	Lobule IX of vermis	3, −60, −39
(4)	Fusiform	Fusiform gyrus + lingual gyrus	Right lingual gyrus	24, −60, −9
(5)	Basal ganglia	Putamen + thalamus + caudate nucleus	Left putamen	−21, 9, −3
(6)	Precentral gyrus	Precentral gyrus + left postcentral gyrus	Left postcentral gyrus	−51, −9, 33
(7)	Anterior DMN	Anterior cingulate gyrus + left medial frontal gyrus	Left medial orbitofrontal cortex	0, 51, −3
(8)	Temporal lobe	Middle temporal gyrus + right superior temporal gyrus	Right superior temporal gyrus	60, −42, 12
(9)	Posterior DMN	Precentral gyrus + left cuneus	Left precuneus	0, −69, 36
(10)	Middle cingulate	Midcingulate area	Left midcingulate area	0, −33, 45
(11)	Occipital medial cortex	Middle occipital gyrus + cuneus + superior occipital gyrus	Right middle occipital gyrus	30, −81, 24
(12)	Frontal superior cortex	Superior frontal gyrus + right supplementary motor area + middle frontal gyrus	Left supplementary motor area	0, 6, 60
(13)	DMN	Medial frontal gyrus	Left medial frontal gyrus	0, 51, 33
(14)	Central gyrus	Postcentral gyrus + precentral gyrus	Undefined	0, −27, 66
(15)	DMN	Middle frontal gyrus + superior frontal gyrus	Right midcingulate area	3, 24, 36
(16)	Precuneus	Precentral gyrus + superior parietal lobule	Left precuneus	0, −57, 54

### 2.4 Machine learning analysis

**Figure 1:**
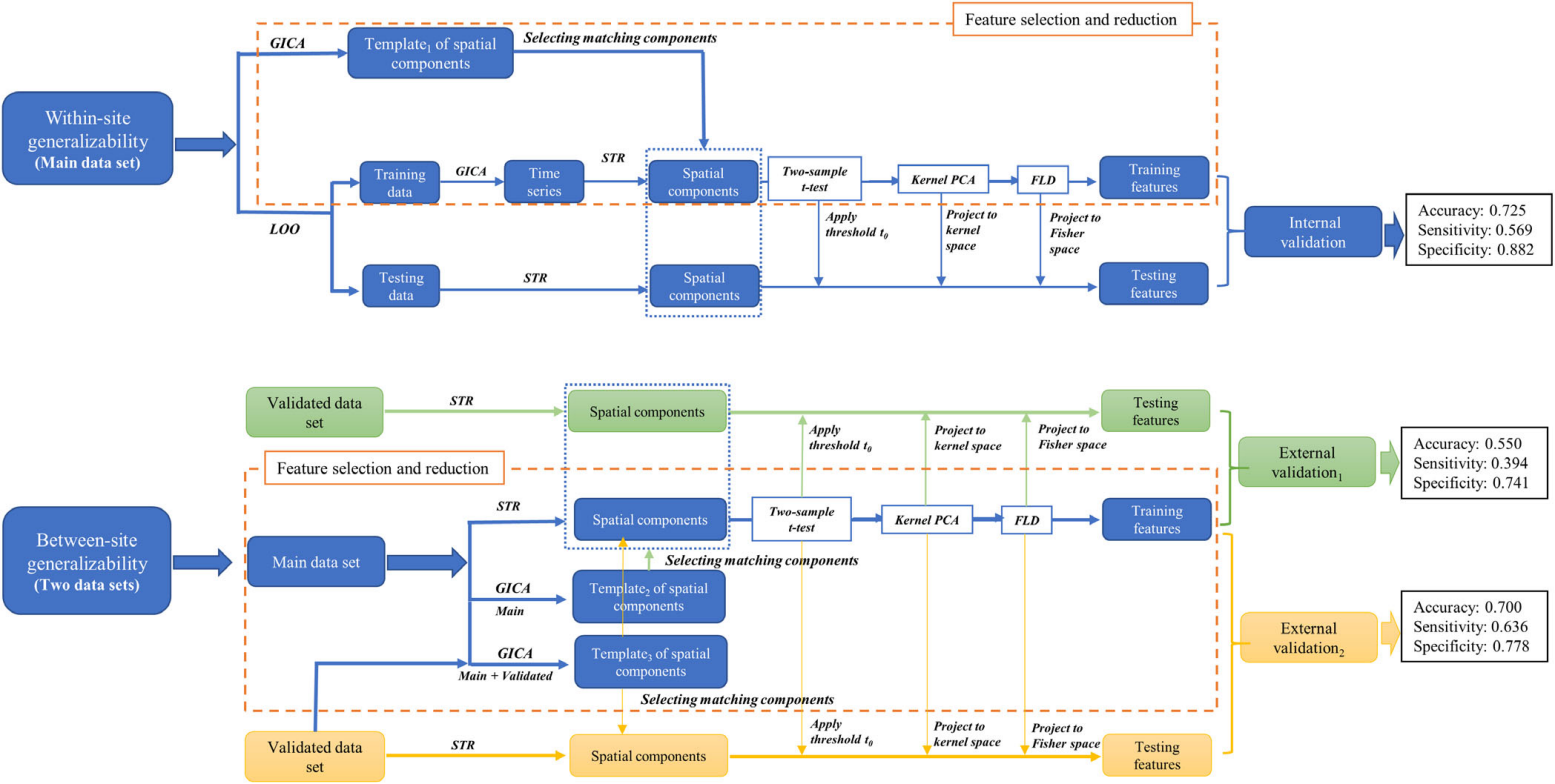
The flowchart shows machine learning procedures through internal and external validation. FLD, Fisher linear discrimination; GICA, group independent component analysis; PCA, principle component analysis; STR, spatial–temporal reconstruction

After preprocessing, further machine learning analysis was conducted (see Figure 1 for the analysis flowchart). To assess generalizability by internal validation, we followed the procedures from Du et al. (2012) in the main data set. To assess between‐site generalizability by external validation, the predictive model built in the main data set was directly applied to the validated data set. To establish transfer learning and to explore factors influencing between‐site generalizability, the unsupervised group independent component analysis (ICA) step was updated based on the two data sets and the between‐site generalizability was estimated again. In general, the machine learning framework consisted of the following steps: (a) extracting and selecting the spatial components (features) for all participants; (b) identification of features with significant differences using a two‐sample *t* test with thresholding; (c) kernel principal component analysis (PCA); (d) Fisher linear discriminant (FLD) analysis on individual components; and (e) majority voting across components.

#### 2.4.1 Assessing within‐site generalizability

In feature extraction and selection, spatial group ICA was first run based on the entire main data set. Data for all participants were concatenated across time. Then, PCA was used to reduce the data into 40 principal components at the participant level. Afterward, ICA was performed to extract 30 independent spatial components based on the resulting data from the PCA at the group level. The analysis of spatial group ICA employed Infomax ICA (Bell & Sejnowski, 1995) in the GIFT software package for MATLAB (GIFT, 2011). Second, the 16 spatial components were selected manually according to the exclusion criteria below:

1. Components originating from artifacts, motion, and respiratory/cardiac cycles, which were identified by considering how much regions known to be associated with these signals were represented in the components. This included edges (motion, respiration), major arteries or veins, the circle of Willis, and the sagittal sinus (cardiac cycle) as well as regions in the vicinity of field inhomogeneity (movement by field inhomogeneity interaction effects).
2. Components consisting of ventricles.
3. Components that showed low and widespread activation.

This step provided a template of spatial components to establish correspondence of components in the subsequent LOO ICA analysis. Then, LOO cross‐validation was used to separate the data into a test set (one participant) and a training set (the remaining sample) and group ICA was performed for the training data. In order to identify individual subject components, spatial–temporal reconstruction (Nickerson, Smith, Öngür, & Beckmann, 2017) based on the ICA model was used to obtain the spatial components for each participant in both the test and training data. A matching procedure based on correlation with the template of spatial components was used to establish component correspondence between the LOO splits. Then, a voxel‐wise two‐sample *t* test between the two groups for the training data was conducted to order the voxel in terms of their *t*‐test value/significance to obtain maximally discriminative features. Only voxels with *t* values larger than a threshold *t*_0_ proceeded to the next step. The threshold *t*_0_ was determined such that the class separation in the final FLD step was maximal within the training data set. When the final threshold value (*t*_0_) was determined, the threshold was applied to the test data. Subsequently, the spatial components for the test and training data were decomposed by kernel PCA. Finally, the test and training data were projected to one dimension by Fisher's linear discriminant analysis (LDA) to make a final prediction of the test label.

After feature extraction and selection, classification was done for each of the 16 components independently and then combined by majority voting to make a final decision on the classification of each test sample. The classification procedure in Du et al.'s (2012) study also reported the classification rates for combinations of components that achieved a high accuracy. However, as this selection procedure might lead to biased classification rates, we only considered classification results based on majority voting across all selected components in the present study.

#### 2.4.2 Assessing between‐site generalizability

To assess between‐site generalizability by external validation, the template of spatial components which was used to select features in assessing within‐site generalizability was applied to the validated set of rsfMRI data. The spatial components for each participant in the validated data set were obtained through spatial–temporal reconstruction based on the template which was built from the main data set. In this setting, determination of the threshold *t*_0_, kernel PCA and training of the LDA classifier considered only the main data set (training set), and hence the identified classification model was directly applied to the validated data set without any adaptation. Apart from changing the LOO cross‐validation to cross‐sample validation, all classification parameters and procedures remained identical.

To further explore factors influencing between‐site generalizability, we updated the ICA step to consider data from both sites. To achieve this, we identified 30 spatially independent components across both data sets using group ICA and established correspondence to the template components from the within‐site generalization procedure using spatial correlation. The matching procedure involved calculating the pairwise spatial correlation between all 30 estimated components and the 16 template components. Then, pairs of components that exhibited the highest absolute correlation were sequentially matched to identify all 16 components in the joint data set. After this matching procedure, the components were visually inspected to confirm that they represented the same brain regions as the template. Importantly, in this setting, the validated data set was only considered in the training of the group ICA model and hence no label information from the validated data set was used in feature extraction or fitting of the classification model. Apart from the different templates of spatial components, the classification procedure was identical to the within‐site generalization setting. The significance of the classification performance was assessed using a random permutation test in all settings where an empirical null distribution was obtained by applying an identical classification procedure to the data with permutated class labels.

## 3 RESULTS

### 3.1 Group ICA on the main rsfMRI data set

**Figure 2:**
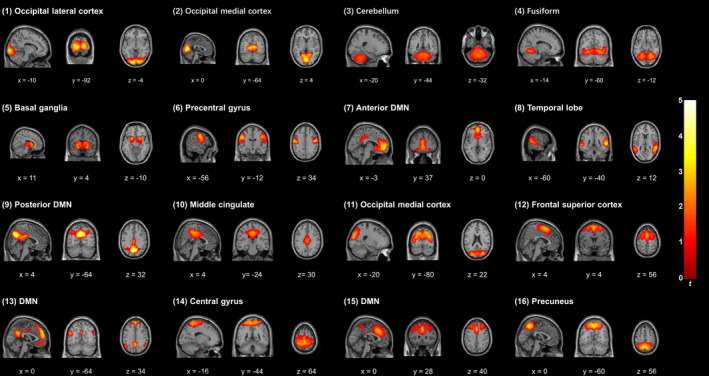
Three orthogonal slices from selected components are shown in the figure. The mean components were calculated across all participants and converted into Z‐scores. Orthogonal projects are reproduced according to neurological convention

Based on the aforementioned criteria, 16 components were selected to classify patients with schizophrenia from healthy controls in the analysis. The selected components are shown in Figure 2. The name, spatial location, peak MNI region, and peak MNI coordinates for each component are shown in Table 5.

**Table 5:** Classification performance for each component

Ind	Area	Validation type	Acc	Sens	Spec	Ind	Area	Validation type	Acc	Sens	Spec
(1)	Occipital lateral cortex	Internal	0.686	0.588	0.784	(9)	Posterior DMN	Internal	0.441	0.392	0.490
		External_1_	0.417	0.485	0.333			External_1_	0.450	0.424	0.481
		External_2_	0.450	0.333	0.593			External_2_	0.583	0.485	0.704
(2)	Occipital medial cortex	Internal	0.578	0.510	0.647	(10)	Middle cingulate	Internal	0.686	0.510	0.863
		External_1_	0.533	0.455	0.630			External_1_	0.433	0.152	0.778
		External_2_	0.583	0.515	0.667			External_2_	0.617	0.636	0.593
(3)	Cerebellum	Internal	0.598	0.392	0.804	(11)	Occipital medial cortex	Internal	0.588	0.353	0.824
		External_1_	0.417	0.333	0.519			External_1_	0.550	0.333	0.815
		External_2_	0.517	0.333	0.741			External_2_	0.550	0.697	0.370
(4)	Fusiform	Internal	0.657	0.490	0.824	(12)	Frontal superior cortex	Internal	0.529	0.627	0.431
		External_1_	0.567	0.364	0.815			External_1_	0.550	0.727	0.333
		External_2_	0.467	0.242	0.741			External_2_	0.533	0.576	0.481
(5)	Basal ganglia	Internal	0.706	0.627	0.784	(13)	DMN	Internal	0.647	0.490	0.804
		External_1_	0.500	0.242	0.815			External_1_	0.483	0.303	0.704
		External_2_	0.667	0.606	0.741			External_2_	0.617	0.576	0.667
(6)	Precentral gyrus	Internal	0.578	0.686	0.471	(14)	Central gyrus	Internal	0.627	0.431	0.824
		External_1_	0.500	0.576	0.407			External_1_	0.617	0.485	0.778
		External_2_	0.633	0.576	0.704			External_2_	0.483	0.242	0.778
(7)	Anterior DMN	Internal	0.618	0.510	0.725	(15)	DMN	Internal	0.431	0.353	0.510
		External_1_	0.600	0.545	0.667			External_1_	0.583	0.606	0.556
		External_2_	0.550	0.788	0.259			External_2_	0.650	0.606	0.704
(8)	Temporal lobe	Internal	0.657	0.667	0.647	(16)	Precuneus	Internal	0.676	0.647	0.706
		External_1_	0.550	0.333	0.815			External_1_	0.550	0.455	0.667
		External_2_	0.667	0.788	0.519			External_2_	0.583	0.333	0.889

*Note*. Ind, index; Acc, accuracy; Sens, sensitivity; Spec, specificity; Internal, internal validation within the main data set; External_1_, external validation generalizing to the validated data set from the main data set by the template based on the main data set; External_2_, external validation generalizing to the validated data set from the main data set by the template based on both data set.

### 3.2 Within‐site classification performance

For classification based on individual features, the performances including accuracy, sensitivity, and specificity of each component are shown in Table 5. We found that the basal ganglia (the fifth feature) achieved the highest accuracy (0.706). The occipital lateral cortex (the first feature), the fusiform gyrus (the fourth feature), the temporal lobe (the eighth feature), the middle cingulate gyrus (the 10th feature), and the precuneus (the 16th feature) achieved a similar accuracy. However, the accuracy of the posterior DMN (the ninth feature) and the DMN (the 15th feature) was lower than random chance. The sensitivity across all features was close to or lower than 0.5, while the specificity across all features was close to or higher than 0.5. When combining all 16 features, majority voting achieved a classification accuracy of 0.725, a sensitivity of 0.569, and a specificity of 0.882.

### 3.3 Between‐site classification performance

**Figure 3:**
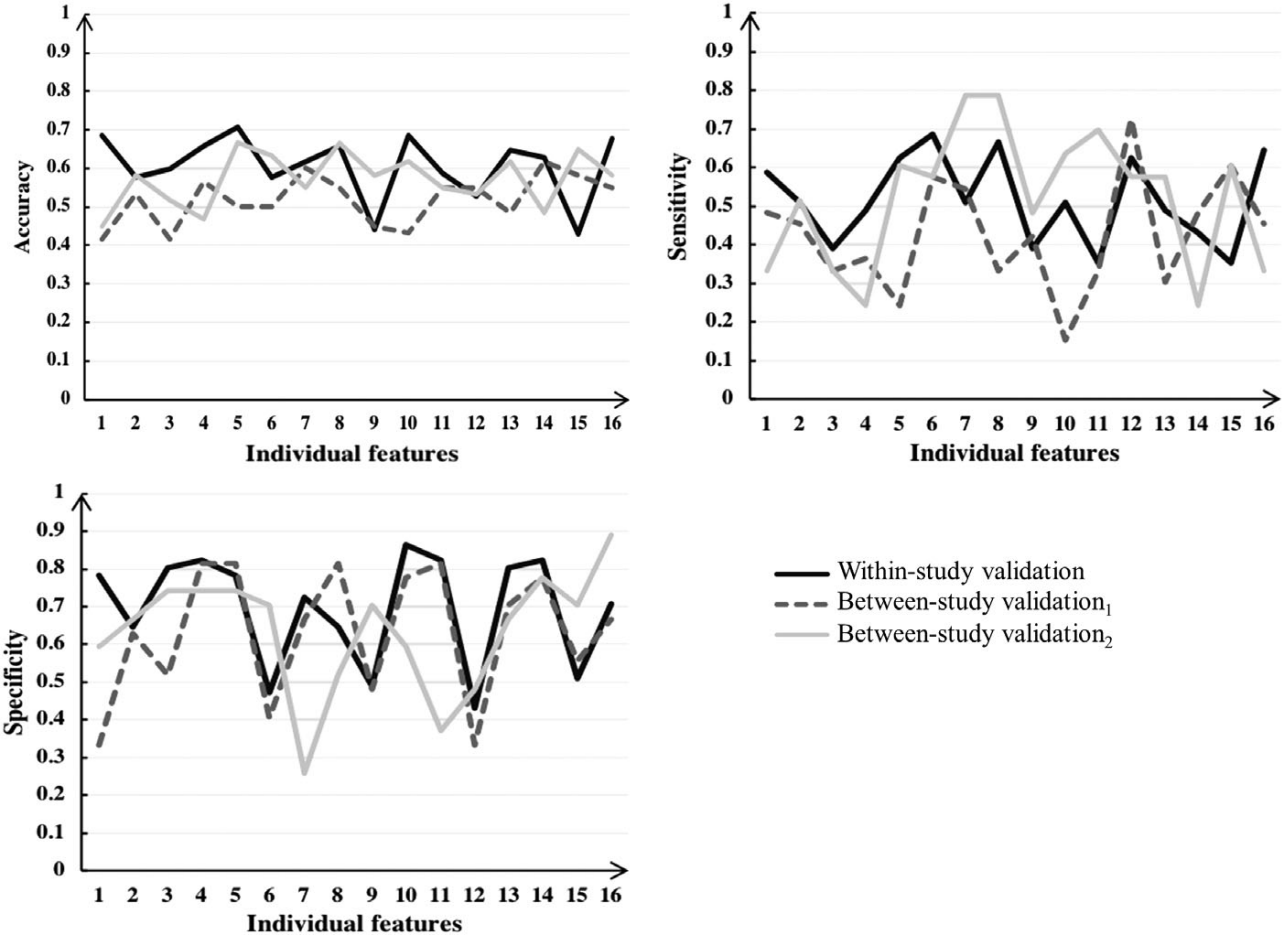
Accuracy, sensitivity, and specificity based on individual features. Internal validation was conducted within the main data set, external validation_1_ was conducted by directly applying the classification procedure from the main data set to the validated data set, and external validation_2_ was conducted using an updated group ICA across both data sets but with all other steps being identical

Between‐site generalizability was assessed using the template of spatial components from the main data set. We found that accuracy in general decreased to the random level for classification from each selected component (see Table 4 and Figure 3). When all 16 features were combined by majority voting, the performance was worse compared to the within‐site performance evaluated by internal validation, with an accuracy of 0.550, a sensitivity of 0.394 and a specificity of 0.741. Random permutation test showed that the accuracy failed to reach statistical significance, with a *p* value of .183.

For the between‐site generalizability by external validation using ICA across both data sets, we found that the performance was similar to the within‐site classification performance for each selected component (see Table 5 and Figure 3). When all 16 features were combined by majority voting, the performance remained similar to the first classification, with an accuracy of 0.700, a sensitivity of 0.636 and a specificity of 0.778. Random permutation test revealed that the accuracy was statistically significant, with a *p* value of .0005.

## 4 DISCUSSION

### 4.1 Classification performance compared with the previous study through LOO

In this study, we first followed a machine learning procedure developed by Du et al. (2012) using rsfMRI data from a sample of schizophrenia patients and healthy controls to assess the within‐site generalizability and the reproducibility of the machine learning procedure. The classification followed the same feature extraction, feature selection, and LOO validation steps as Du et al. (2012). We found that based on individual features, the highest classification accuracy was 0.706, while the classification accuracy was 0.725 when all selected components were combined. To establish correspondence with Du et al.'s (2012) study, the performance of individual components can be found in Table 5. Since testing individual components or combinations of those would result in multiple comparisons and the classification results using individual components were less stable than the result from majority voting, we focused on the results obtained from majority voting across all 16 components to avoid overinterpretation. Although the accuracy in the present study is not as high as the results from Du et al. (2012), compared with the overall reported accuracy of 65–95% in the field (Anderson & Cohen, 2013; Bassett et al., 2012; Fan et al., 2011; Su et al., 2013; Venkataraman et al., 2012), our results support the reproducibility of the machine learning procedure which can successfully discriminate patients with schizophrenia from healthy controls.

Several factors may explain the reduced within‐site generalizability we found. First, 51 pairs of participants were recruited for LOO validation in our study, but Du et al.'s study only had 28 pairs. The smaller sample size in Du et al.'s study in the context of analyzing high‐dimensional imaging data could easily lead to overfitting of the predictive model to their data set (Mwangi, Tian, & Soares, 2014). Second, the longer illness duration and lower symptom severity of our patients might have introduced more heterogeneity into the profile of participants (an der Heiden & Hafner, 2000; Irani, Kalkstein, Moberg, & Moberg, 2011), making it more difficult to identify patterns that are directly related to the disorder in question. Finally, although comprehensive preprocessing was performed to reduce noise and limit the effect of artifacts on the analysis (Churchill et al., 2011), these confounding factors could still persist and could affect the classification performance.

### 4.2 Implication of between‐site generalizability

When assessing between‐site generalizability in two completely independent data sets from two sites, we obtained a nonsignificant accuracy of 0.550 and a significant accuracy of 0.700 based on all selected features when applying a template of spatial components built based on only the main data set and both data sets, respectively. It is important to note that although the validated data set was utilized with the main data set for generating a new template of spatial components through spatial group ICA, the labels of the validated data set were still kept unseen when training the model to ensure that the training and testing data sets were independent.

More importantly, the findings that the predictive model did not successfully generalize to the novel data set when applying the template only based on the training data set but successfully generalized to the independent testing data set when applying the template based on both data sets substantially extends our understanding of the generalizability of machine learning using rsfMRI data. Failure to generalize does not necessarily mean that a model is invalid, since multiple factors are different between independent data sets (Scheinost et al., 2019). As indicated in Table 3, the schizophrenia patients in the main data set were significantly older, had significantly higher estimated IQ, longer illness duration and higher PANSS positive subscale score, but significantly lower PANSS total, negative and general subscale scores compared with patients in the validated data set. In addition, the healthy controls in the main data set had significantly higher estimated IQ than the healthy controls in the validated data set. Such heterogeneity between the two data sets may be one potential factor contributing to the failure in direct generalization. In addition, the two data sets were acquired independently from different scanners with different acquisition parameters, which could further compromise generalizability. The differences in classification performance highlights the importance of considering differences in the new data when constructing classification models and demonstrates the usefulness of unsupervised transfer learning in this setting. Moreover, meta‐analytic studies of machine learning in schizophrenia have demonstrated that age, medication exposure, illness stage, and gender are all significant moderators of classification performance (Kambeitz et al., 2015; Neuhaus & Popescu, 2018).

Cui, Liu, Song, et al. (2018) and Cui, Liu, Wang, et al. (2018) evaluated the classification performance of their machine learning algorithm within the same data set and across different data sets and also found that the classification accuracy across different data sets was lower than within the same data set. However, the extent of difference in accuracy was not large in their study compared with ours. This could be due to differences in participant characteristics. In their study, patients with untreated first‐episode schizophrenia were recruited and the demographics were very similar between the two samples; whereas in our study, we recruited patients with chronic schizophrenia and the demographics were different between the two samples. Taken together, these findings suggest that future research to construct machine learning models should take into account illness heterogeneity.

### 4.3 Implication for schizophrenia research

The classification performance based on individual features in this study revealed that the striatum yielded the highest accuracy. Spatial components including the lateral occipital cortex, the fusiform gyrus, the temporal lobe, the middle cingulate gyrus, the DMN, and the precuneus could also distinguish patients with schizophrenia from healthy controls with acceptable accuracy. These findings are supported by previous studies using machine learning methods in schizophrenia (Du et al., 2012; Fan et al., 2011; Savio & Grana, 2015; Tang et al., 2012; Yu et al., 2013). Our findings suggest that the striatum may play a key role in schizophrenia. This is consistent with results from previous studies which demonstrated alteration in striatal volume (Chakravarty et al., 2015) and white matter connectivity (James et al., 2016). For the DMN, studies by Fan et al. (2011) and Du et al. (2012) also suggest that it is one of the most informative brain regions for the diagnosis of schizophrenia. Moreover, a recent study found that the DMN interacted with the central executive network and the salience network in smoking schizophrenia patients, indicating the potential role of the DMN in the symptomatology of the disorder (Liao et al., 2018). At the same time, the structural and functional alterations in the DMN in schizophrenia patients have been shown to be related to impairment in working memory and attention (Garrity et al., 2007; Hu et al., 2017; Pomarol‐Clotet et al., 2008; Salgado‐Pineda et al., 2011; Whitfield‐Gabrieli & Ford, 2012). Previous studies have also suggested that the fusiform gyrus and the temporal lobe may be discriminative features for classification (Savio & Grana, 2015; Tang et al., 2012). In anatomical studies, reduced gray matter volume at the superior temporal gyrus has been reported in schizophrenia patients (Ohi et al., 2016; Sun, Maller, Guo, & Fitzgerald, 2009), which may also be related to hallucinations (Cui, Liu, Song, et al., 2018; Cui, Liu, Wang, et al., 2018).

### 4.4 Limitation

This study has several limitations. First, the sample size in the present study was small. Bigger data sets are needed to avoid overfitting and to build a classifier with better generalizability. Second, only one modality of data (rsfMRI) was utilized, even though both functional and structural brain information may be important for high‐accuracy classification using machine learning (Mikolas et al., 2017; Orban et al., 2018; Ota et al., 2012). Third, we did not collect the smoking status of our participants in this study. Considering the high rate of smoking in schizophrenia patients (Liao et al., 2018), which may influence brain activity (Potvin et al., 2016; Tanabe, Tregellas, Martin, & Freedman, 2006), smoking status may be a confounding factor that affects classification performance. Fourth, since our findings suggest the importance to take scanning setting and characteristics of participants into account when collecting data from different sites, future studies should examine how these two sets of factors affect between‐site generalizability.

## 5 CONCLUSION

In this study, we found that the machine learning procedure developed by Du et al. (2012) could successfully classify patients with schizophrenia from healthy controls based on rsfMRI data in internal validation but not external validation. Moreover, we found that a transfer learning procedure based on unsupervised learning was able to improve between‐site generalizability and may eventually contribute to the incorporation of machine learning approaches into clinical practice.

## CONFLICT OF INTEREST

The authors declare no potential conflict of interest.

## Data Availability

The data of the present study are available from the corresponding author upon request.
